# A g_*m*_/I_*D*_-Based Low-Power LNA for Ka-Band Applications

**DOI:** 10.3390/s24082646

**Published:** 2024-04-21

**Authors:** David Galante-Sempere, Jeffrey Torres-Clarke, Javier del Pino, Sunil Lalchand Khemchandani

**Affiliations:** Institute for Applied Microelectronics (IUMA), Universidad de Las Palmas de Gran Canaria, 35001 Las Palmas de Gran Canaria, Spain; jtorres@iuma.ulpgc.es (J.T.-C.); jpino@iuma.ulpgc.es (J.d.P.); sunil@iuma.ulpgc.es (S.L.K.)

**Keywords:** low noise amplifier, cascode, low-power, g_*m*_/I_*D*_, Ka band, 45 nm, silicon-on-insulator

## Abstract

This article presents the design of a low-power low noise amplifier (LNA) implemented in 45 nm silicon-on-insulator (SOI) technology using the $g_{m}$/$I_{D}$ methodology. The Ka-band LNA achieves a very low power consumption of only 1.98 mW andis the first time the $g_{m}$/$I_{D}$ approach is applied at such a high frequency. The circuit is suitable for Ka-band applications with a central frequency of 28 GHz, as the circuit is intended to operate in the n257 frequency band defined by the 3GPP 5G new radio (NR) specification. The proposed cascode LNA uses the $g_{m}$/$I_{D}$ methodology in an RF/MW scenario to exploit the advantages of moderate inversion region operation. The circuit occupies a total area of 1.23 mm^2^ excluding pads and draws 1.98 mW from a DC supply of 0.9 V. Post-layout simulation results reveal a total gain of 11.4 dB, a noise figure (NF) of 3.8 dB, and an input return loss (IRL) better than 12 dB. Compared to conventional circuits, this design obtains a remarkable figure of merit (FoM) as the LNA reports a gain and NF in line with other approaches with very low power consumption.

## 1. Introduction

Low noise amplifiers (LNAs) are the first active components in the analog front end of any conventional receiver and are generally considered one of the most power-hungry blocks, as their performance is critical for the overall system. The LNA dictates the receiver’s noise figure (NF) and sensitivity [1,2,3]. The LNA’s high power consumption stems from the fact that it must provide adequate input matching, high gain, low noise, and high linearity simultaneously, all of which require high power as well as high supply voltages. These combined specifications have made the design of low-power and low-voltage LNAs a challenging research topic [4,5,6,7,8,9,10]. Given a certain circuit topology, the conventional LNA design approach consists of finding the optimal current density for minimum NF, maximum gain, or covering the application requirements. It requires several iterations to obtain a successful design covering the desired specifications and relies heavily on the designer’s expertise and intuition. A very attractive methodology to exploit the advantages of subthreshold MOSFET operation, reduce the time-consuming design flow, and efficiently explore the LNA design space is the $g_{m}$/$I_{D}$ methodology [11,12,13,14]. It explores the ratio between the small-signal transconductance ($g_{m}$) of a MOSFET and the DC drain current ($I_{D}$), known as the MOSFET efficiency. The $g_{m}$/$I_{D}$ methodology has been widely used in analog integrated circuit designs to obtain very-low-power circuits for relatively low-frequency applications [4,6,15,16,17,18,19]. The main advantage of the methodology is that it provides a powerful sizing tool that allows the designer to take advantage of all the subthreshold regions to obtain very low power consumption circuits with very few iterations and significant time reduction in the design flow. As demonstrated by [11,12], by studying the DC bias conditions and small-signal characteristics of the MOSFETs in a PDK, circuit designers can generate a series of look-up tables (LUTs) with all the information needed to obtain a circuit given a target specification. The main principle of the methodology is the use of a device-independent parameter (frequently, a figure of merit such as the inversion coefficient or the $g_{m}$/$I_{D}$ ratio) as the main design parameter to explore the design space or determine the optimal operation region. Thanks to the generated LUTs, the need for iterative simulations is removed and near-first-time success can be achieved to cover the design requirements. Although the $g_{m}$/$I_{D}$ methodology was born in the field of low-frequency analog integrated circuit design, significant efforts have been made to integrate this methodology with radiofrequency (RF) and microwave (MW) circuit design [14,20,21,22,23], demonstrating the implementation of very-low-power RFICs. However, these proposals are limited to operating frequencies of only 2.4 GHz, with no $g_{m}$/$I_{D}$-based LNA designs above an operating frequency of 5 GHz reported.

In contrast to traditional bulk silicon (Si) complementary metal-oxide-semiconductor (CMOS) processes, silicon-on-insulator (SOI) technologies present multiple advantages, such as reduced parasitic capacitances, higher quality factor (Q, figure of merit related to passive components’ narrow-band response and insertion loss), improvement of device performance and speed, downsizing to nanometer dimensions, reduction in device operating voltage, lower power consumption, and reduced leakage currents, among others [24]. Although III–V compounds such as gallium arsenide (GaAs) technologies are generally employed in very-high-frequency scenarios with demanding NF requirements such as mm-wave applications, 5G networks, or satellite communications (SATCOMs), SOI technologies provide a comparable performance while facilitating system integration with lower production costs. Both III–V and SOI technologies can find application in high-resolution radar, short-range military aircraft radios, and astronomical observations, which operate at Ka-band frequencies [25,26,27,28,29]. For instance, the 28 GHz frequency band is identified as a pioneer band to host 5G new radio (NR) networks worldwide. It provides a very high data rate and capacity, making it a convenient choice for hotspot coverage. In this sense, the European Union designated the n258 and n257 frequency bands, which extend from 24.25 to 27.5 GHz and 27.5 to 29.5 GHz, respectively, the US identified the 27.5–28.35 GHz band, and Japan and Korea considered the 27.5–29.5 GHz and 26.5–29.5 GHz bands, respectively, for the same purpose [30]. Therefore, the developments introduced in Ka-band LNAs greatly benefit 5G mm-wave systems and applications all over the world.

In this work, we apply the $g_{m}$/$I_{D}$ methodology, adapted to an RF/MW environment, to obtain a Ka-band very-low-power LNA with a remarkable performance thanks to the advantages introduced by exploiting the moderate inversion region of a MOSFET. The proposed LNA achieves a very low power consumption of only 1.98 mW and is the first time the $g_{m}$/$I_{D}$ approach is applied at such a high frequency. The 28 GHz cascode LNA is implemented in GlobalFoundries 45nm RFSOI and occupies an area of 1.23 mm^2^ excluding pads. The circuit achieves a gain of 11.4 dB with an NF of 3.8 dB and input return loss (IRL) better than 12 dB when fed from a 0.9 V DC supply, drawing only 1.98 mW. The organization of this paper is the following. Section 2 presents the circuit design of the proposed $g_{m}$/$I_{D}$-enabled LNA. Section 3 shows the post-layout simulation results and Section 4 concludes this paper.

## 2. Design Procedure

**Figure 1:**
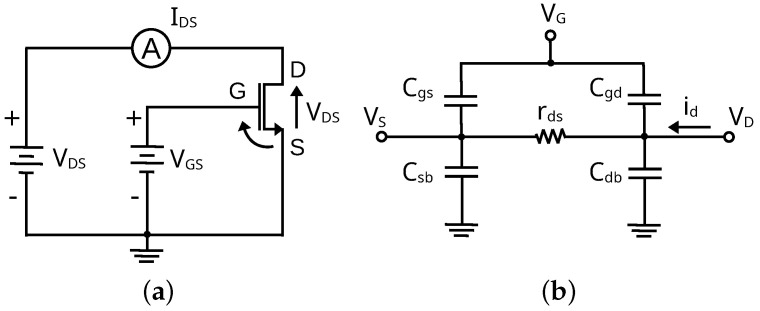
Schematic diagram of the setup used to obtain the I−V curves of the FB−FET from GlobalFoundries 45RFSOI PDK (**a**) and representation of the device capacitances (**b**).

The $g_{m}$/$I_{D}$ design procedure is a powerful sizing and biasing tool for MOSFET-based circuits [11,12]. The methodology exploits the ratio between the transconductance and the drain current as they are both width-dependent parameters to obtain a width-independent design variable. Since this ratio gives the ability of a MOSFET to generate a small-signal current gain from a DC bias current, the $g_{m}$/$I_{D}$ ratio is often referred to as the MOSFET efficiency. The characterization of a MOSFET in terms of $g_{m}$/$I_{D}$ ratio allows the derivation of the main performance metrics of a circuit to provide a width-independent sizing tool, allowing fast optimization and near-first-time successful design. A series of LUTs can be generated to avoid conventional SPICE iterative simulations, and designers can apply optimization algorithms to find optimal sizing and operating points or simply explore the design space more efficiently [31]. The first step consists of simulating several devices to fully characterize their behavior and build the LUTs with fundamental DC, AC, and noise parameters. These values can then be used to design LNAs given the specifications and circuit topology. The floating-body (FB) transistors of the GlobalFoundries 45RFSOI process design kit (PDK) are chosen to design the LNA. Since they are FB transistors, they do not possess substrate contact. To analyze their behavior, several geometries are simulated to fully characterize the behavior of the FETs in the selected kit. The total width (W) is varied from 20 to 120 μm in steps of 5 μm; the length (L) comes in discrete values of 32, 40, and 48 nm; and the unit finger width ($w_{f}$) is varied from 0.5 to 2 μm, which is the maximum range available in the PDK. In addition, the DC voltage $V_{GS}$ is varied from 0 V to 1 V in steps of 25 mV and $V_{DS}$ is varied from 0 V to 1 V in steps of 25 mV, using the schematic shown in Figure 1a, which is used to simulate all the parameters analyzed in this section.

The DC parameters observed include the drain current $I_{DS}$, the threshold voltage $V_{TH}$, the gate-drain current $I_{GD}$, and the gate-source current $I_{GS}$. On the other hand, the AC parameters obtained are regular transconductance $g_{m}$, gate-bulk transconductance $g_{mb}$, drain-source transconductance $g_{ds}$, and all FET capacitances (shown in Figure 1b, gate-source $C_{gs}$, gate-drain $C_{gd}$, source-bulk $C_{sb}$, drain-bulk $C_{db}$). Finally, the noise of the FET is characterized using two main parameters: STH and SFL (thermal and flicker noise, respectively).

To automate the sweeps and generate the LUTs efficiently, the simulations are automated using a single MATLAB script that performs the following tasks:

- Generates DC sweeps for all corners. These corners are generated by considering all possible combinations of process, temperature, and noise variations and depend on the PDK used. In this case, the combinations include typical (T), slow (S), and fast (F) devices with −40 °C, 16 °C, 125 °C and high-, nominal-, and low-noise corners.
- Maps the FET’s operating point parameters into the desired output variables to build a multidimensional MATLAB matrix from the Cadence database results.
- Generates the Spectre simulation netlists with the desired geometries and sweeps.
- Sequentially runs all the previously generated simulations.
- Generates a .mat file with the multidimensional data for each corner as a result.

The original code provided by [11,12] is adapted to accommodate the 45RFSOI PDK and the mentioned sweeps. A simple *lookUp()* function is then used to recover the desired values and plot the desired parameters. More information about this process can be found in [11,12].

**Figure 2:**
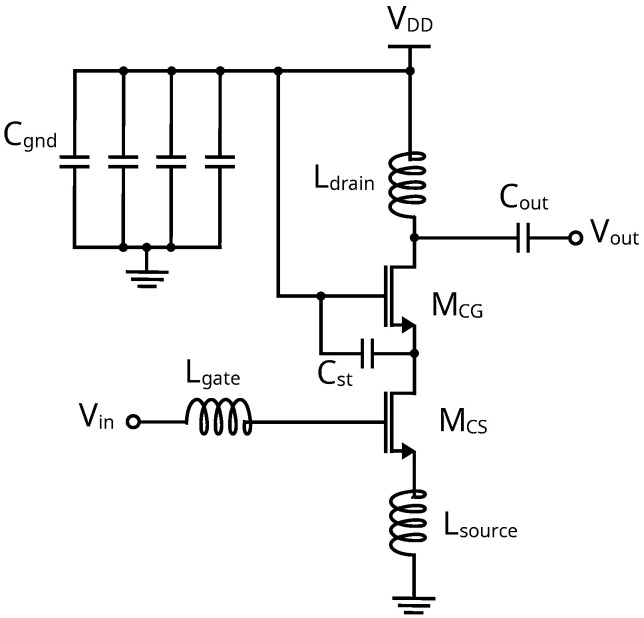
Schematic diagram of the cascode LNA developed.

Since the Ka-band frequency range spans from 26.5 GHz to 40 GHz and the n257 frequency band (26.5 to 29.5 GHz) defined in the 3GPP 5G NR specification is particularly interesting for European mm-mave communications, a central frequency of 28 GHz is considered for the LNA design. To achieve high gain with a low NF and reasonable power consumption, a cascode topology, as shown in Figure 2, is selected [32,33,34,35,36]. It provides a high output impedance and higher input/output isolation compared to common-source and common-gate amplifiers, which allows the designer to cascade several stages if a higher gain is needed.

The inversion coefficient (IC) is used to identify the sub-threshold operation region of a MOSFET and is expressed using Equation (1), with W/L being the MOSFT aspect ratio and $I_{spec□}$, the specific current defined as (2) [37]. Details regarding the obtaining of $I_{spec□}$ for a given process can be found in [37]. The definition of IC results in the definition of three inversion regions: weak inversion (for IC≤0.1), moderate inversion (for 0.1<IC≤10), and strong inversion (10<IC).

(1) $$IC=\frac{I_{DS}}{I_{spec□}\cdot W/L}$$

(2) $$I_{spec□}=2n\mu_{0}C_{ox}U_{T}^{2}$$

Following [14], a figure of merit for RF performance (${\mathrm{FoM}}_{RF}$) can be defined, as shown in Expression (3), which can be employed to find the optimal inversion coefficient IC value for a given transistor in a high-frequency design.

(3) $$FoM_{RF}=\left(g_{m}/I_{D}\right)\cdot f_{T}$$

**Figure 3:**
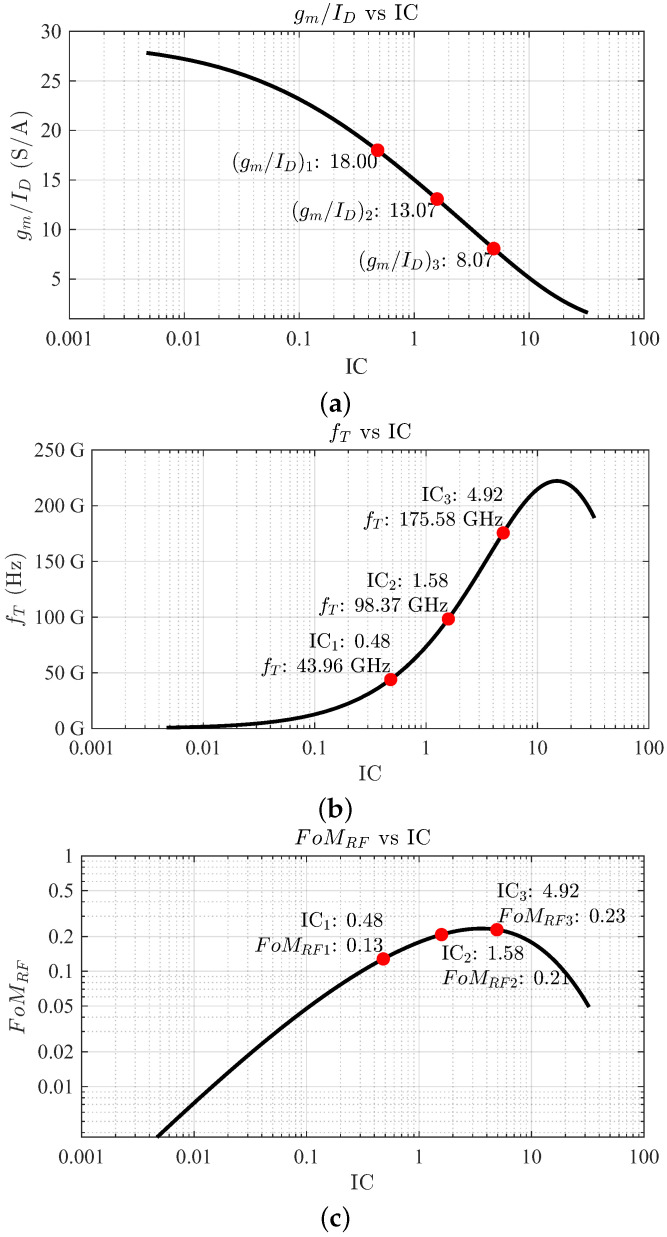
Representation of $g_{m}$/$I_{D}$ (**a**), $f_{T}$ (**b**), and ${\mathrm{FoM}}_{RF}$ (**c**) as functions of the inversion coefficient IC.

The values of $g_{m}$/$I_{D}$, $f_{T}$, and ${\mathrm{FoM}}_{RF}$ are presented in Figure 3a, Figure 3b and Figure 3c, respectively. Note that, as seen in Figure 3a, $g_{m}$/$I_{D}$ is maximal in the weak inversion region and it decreases as IC moves toward the strong inversion region. On the other hand, the $f_{T}$ value (Figure 3b) is remarkably low in weak inversion and it rises as IC moves towards strong inversion. The result, as expressed in (3) and presented in Figure 3c, is that the moderate inversion region achieves the optimal trade-off and it benefits from the best combination of transistor efficiency $g_{m}$/$I_{D}$ and high-frequency performance ($f_{T}$). However, with this approach, the value of the input impedance ($Z_{11}$) and optimal NF impedance ($Z_{opt}$) are not known yet. Impedance selection is critical in the design process, as the input matching network implementation severely affects the gain and noise performance of the LNA.

In conventional cascode amplifier design, where a common-source and a common-gate amplifier are used in series, simultaneous minimum NF and maximum gain matching can be achieved if source degeneration ($L_{S}$) is applied. Generally, the drain current density and transistor width are increased to move $Z_{opt}$ to the 50 Ω circle, and then a single gate inductance ($L_{G}$) can be employed to match the circuit [38]. Therefore, a $Z_{opt}$ with a real part close to 50 Ω is desired to facilitate impedance matching with a single gate inductor. To consider device geometries that allow this condition, assume the input impedance of a CS amplifier is given as (4) and the real part of the optimum source impedance is (5) [38,39]. From the state of the art, at Ka-band frequencies, an $L_{G}$ under 500 pH and an $L_{S}$ between 50 and 250 pH are conventionally used. Notice the parameters in (5) are known, and thus, the required width for $Z_{opt}$ = 50 Ω can be obtained.

(4) $$Z_{in}=r_{g}+s\left(L_{G}+L_{S}\right)+\frac{1}{sC_{g}s}+\frac{g_{m}L_{S}}{C_{g}s}$$

(5) $$Re\left[Z_{opt}\right]\approx \sqrt{\frac{r_{g}}{2g_{m}}}\times \frac{f_{T}}{f}$$

**Figure 4:**
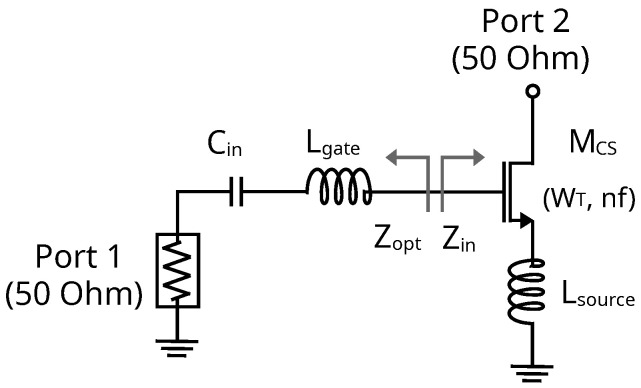
Schematic used to obtain the input and optimal NF impedances.

A total device width close to 50 μm is determined to satisfy the condition of an $Z_{opt}$ with a real part close to 50 Ω. To provide some insights into the impedances defined in (4) and (5), a graphical representation is more clearly reflected in Figure 4. Proper choice of the source inductor value can ensure that $Z_{opt}$ and $Z_{in}$* are approximately equal for maximum power transfer. Then, a single gate inductor can be used to cancel the capacitive component at the gate of $M_{CS}$ in Equation (4).

For the selected technology and the transistor employed, the total width value is determined to be 50 μm. The design approach consists of selecting a certain IC value that accommodates the specifications of the LNA. To decide which IC should be used, consider the following discussion. The transit frequency $f_{T}$ limits the frequency of operation and factors such as the achievable gain and NF [38]. The $f_{T}$ is closely related to the IC, as shown in Figure 3b. If the transistor’s $f_{T}$ is too close to 28 GHz, the LNA may not achieve a reasonable performance (low gain and high NF), but it may have low power consumption, as the lower the IC, the lower the drain current needed to bias the device. On the other hand, if the $f_{T}$ is very high (*n* times the operating frequency), the LNA will offer very high performance (high gain and low NF), but with high power consumption.

**Figure 5:**
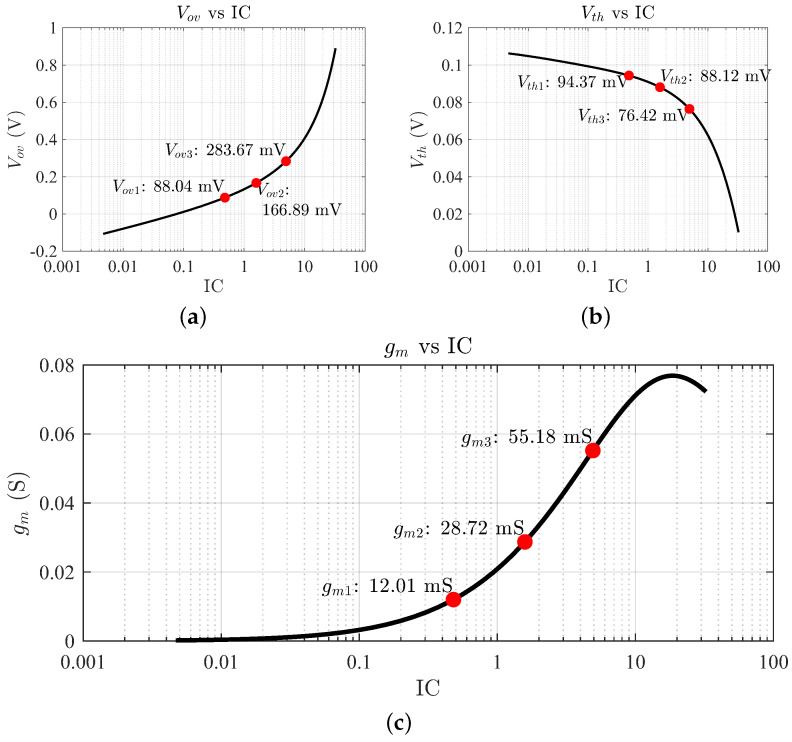
Simulation results of the overdrive voltage $V_{ov}$ (**a**), threshold voltage $V_{TH}$ (**b**), and transconductance (**c**) as functions of IC.

The advantage of using the $g_{m}$/$I_{D}$ methodology is that the designer can access the LUTs and produce several sets of values to perform several designs. As an example to carry on with the design, consider three values: $f_{T1}=$ 44 GHz, $f_{T2}=$ 98 GHz, and $f_{T3}=$ 175 GHz. Since the value of $f_{T}$ is approximately given by (6) for a MOSFET and $g_{m}$ increases as does $I_{DS}$, the $f_{T}$ increases as the IC is augmented. The moderate inversion region is targeted for 44 GHz, the moderate-strong inversion region for 98 GHz, and the strong inversion region for 175 GHz. Operation in weak inversion results in a very high aspect ratio (W/L), and, therefore, the device presents significant capacitance with low drain current, resulting in poor high-frequency operation. The proof-of-concept design process is conducted for these $f_{T}$ values to make a comparison and select the best performance compromise. From Figure 3b, the value of IC is deduced, which is needed to obtain the DC operating point from the LUTs. In the case of the 44 GHz frequency, the corresponding ${\mathrm{IC}}_{1}$ value is 0.48. For 98 GHz, ${\mathrm{IC}}_{2}$ has a value of 1.58, and for 175 GHz, ${\mathrm{IC}}_{3}$ has a value of 4.92. The next step is to calculate the values of threshold voltage ($V_{TH}$) and effective gate-source voltage or overdrive voltage ($V_{GSeff}$ or $V_{ov}$) to properly bias the MOSFET. These parameters are plotted as a function of IC, as illustrated in Figure 5a,b.

(6) $$f_{T}=\frac{g_{m}}{2\pi (C_{gs}+C_{gd})}$$

The required $V_{GS}$ voltage for the transistor to operate with the desired IC can be deduced from the previous figures and is given by Equation (7). $V_{GS}$ sets the desired current flowing through the FETs, and thus, the higher the IC, the higher the $V_{GS}$ needed to bias the device with the desired drain current. For the 44 GHz $f_{T}$ case, $V_{GS1}$ has a value of 182.41 mV to obtain a drain current of 0.67 mA; for the 98 GHz case, $V_{GS2}$ is 255 mV for $I_{D}$ = 2.2 mA; and for the 175 GHz case, $V_{GS3}=$ 360 mV to set an $I_{D}$ of 6.8 mA.

(7) $$V_{ov}=V_{GS}-V_{TH}$$

To fully characterize the FETs, the transconductances $g_{m}$ for each IC are collected, which determine the transistor capability to produce a drain current change from an increment in $V_{GS}$. As shown in Figure 5c, an ${\mathrm{IC}}_{1}$ of 0.48 yields a $g_{m1}$ of 12 mS; for ${\mathrm{IC}}_{2}$ = 1.58, $g_{m2}$ is 28.72 mS; and for ${\mathrm{IC}}_{3}$ = 4.9, $g_{m3}$ is 55.18 mS. As expected, the transconductance increases as does the drain current and is maximal in the strong inversion region. Similarly, the transistor efficiency ($g_{m}$/$I_{D}$) versus IC is sought, as shown in Figure 3a. As opposed to the transconductance, the transistor efficiency is usually maximal in weak inversion and it decreases as the operation region moves towards strong inversion; hence, for ${\mathrm{IC}}_{1}$, ${\left(g_{m}/I_{D}\right)}_{1}$ is 18 S/A; for ${\mathrm{IC}}_{2}$, ${\left(g_{m}/I_{D}\right)}_{2}$ is reduced to 13.07 S/A; and for ${\mathrm{IC}}_{3}$, ${\left(g_{m}/I_{D}\right)}_{3}$) is only 8.07 S/A. With the values of $g_{m}$/$I_{D}$ and $g_{m}$ available, the drain current $I_{D}$ can be solved from Equation (8).

(8) $$I_{D}=\frac{g_{m}}{g_{m}/I_{D}}$$

**Figure 6:**
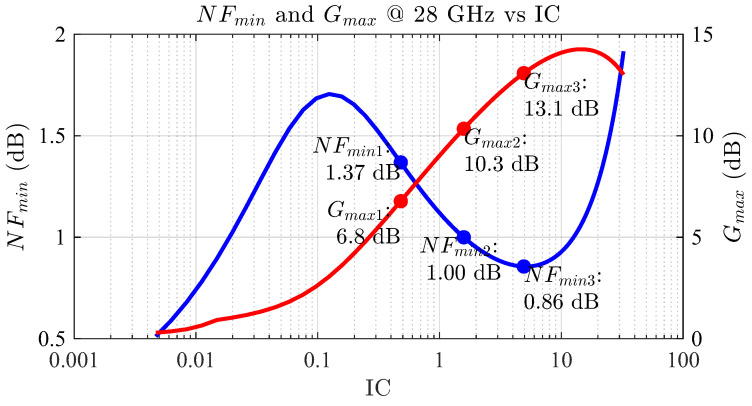
Maximum gain $G_{max}$ and ${\mathrm{NF}}_{min}$ as a function of the inversion coefficient.

At this point, the designer may wonder about the actual devices’ high-frequency performance for the selected IC values. Therefore, the $G_{max}$ and ${\mathrm{NF}}_{min}$ can be calculated, as shown in Figure 6. The case of ${\mathrm{IC}}_{1}$ suffers from a limited $G_{max1}$ of 6.78 dB due to the lower $f_{T}$ and $g_{m}$, as well as an increased ${\mathrm{NF}}_{min1}$ of 1.37 dB for the same reason. Note these are ideal values assuming ideal matching networks are used and are expected to deviate to some extent once real components are added to the circuit. That means the actual circuit implementation with PDK components results in a higher NF and a lower gain due to finite Q factors and parasitic components. For the case of ${\mathrm{IC}}_{2}$, the $G_{max2}$ is significantly improved to 10.34 dB because of the increase in both $f_{T}$ and $g_{m}$, and ${\mathrm{NF}}_{min2}$ in this case is 1 dB. Finally, for the case of ${\mathrm{IC}}_{3}$, the $G_{max3}$ obtained is 13.08 dB and ${\mathrm{NF}}_{min3}$ is as low as 0.86 dB. Since the original setup collects data from a single transistor, e.g., a common-source amplifier, both a higher gain and NF are expected when the cascode is set up.

The advantage of using this methodology is that LNAs can be designed by optimizing the power consumption for given specifications without the need for iterative simulations. Instead, a database with transistor parameters for a specific technology is available. In summary, Table 1 presents the values of the calculated parameters for the three IC values of 0.48, 1.58, and 4.92.

Table 1 illustrates the design trade-offs mentioned earlier. The most favorable among the three cases can be determined through the analysis of power consumption and a figure of merit (${\mathrm{FoM}}_{IC}$), as defined by Equation (9). Depending on the desired characteristics, the corresponding FoM definition and inversion region should be chosen. For instance, for our definition of ${\mathrm{FoM}}_{IC}$, if low power consumption is desired, the weak inversion region should be selected. However, this choice comes with the flaw of increased ${\mathrm{NF}}_{min}$ and a lower $G_{max}$ compared to the other cases. On the other hand, if a high gain with a lower minimum noise figure is sought, it should be noted that the required $I_{D}$ increases, leading to higher power consumption. In contrast to low-frequency designs, in this case, the transistor area is not particularly relevant, as the impedance-matching components (inductors) occupy most of the space. Their area could be included in the definition of the FoM if the designer wants to account for them in a fairer comparison. These parameters enable us to assess and compare the quality of the different cases.

As shown in Table 1, in the case of ${\mathrm{IC}}_{1}$, a high value of the ${\mathrm{FoM}}_{IC}$ is achieved with very low power consumption, but with unfavorable results, the NF is significantly high and the gain is under 10 dB. However, the ${\mathrm{FoM}}_{IC1}$ = 5.7 indicates that the LNA is more efficient in generating a high gain and low NF performance from the current drawn. For ${\mathrm{IC}}_{1}$, the LNA shows a maximum gain of 8.6 dB and an ${\mathrm{NF}}_{min}$ of 3.9 dB, but it draws only 0.67 mA. Whereas the power consumption of solution ${\mathrm{IC}}_{1}$ is remarkable, the gain and NF values are not in line with state-of-the-art K-band LNA designs. A gain above 10 dB and NF under 3 dB with ∼10 mA are considered state-of-the-art results. Notice the results for ${\mathrm{IC}}_{2}$, with a $G_{max}$ increment of more than 5.5 dB compared to ${\mathrm{IC}}_{1}$ (to 14.2 dB) and ${\mathrm{NF}}_{min}$ reduction of 2 dB (to 1.9 dB), with a three-times-higher drain current (2.2 mA) required compared to ${\mathrm{IC}}_{1}$. Considering similar works available in the literature, these values of $G_{max}$ and ${\mathrm{NF}}_{min}$ are closer to state-of-the-art K-band LNAs. As seen for ${\mathrm{IC}}_{3}$, the values of $G_{max}$ and ${\mathrm{NF}}_{min}$ are improved further to 17 dB and 1.4 dB, respectively, but the current drawn increases as well. The required $I_{D}$ is three times that required to bias the ${\mathrm{IC}}_{2}$ LNA, yet the ${\mathrm{NF}}_{min}$ is only improved by 0.5 dB, and the maximum gain, by less than 3 dB. In the case of ${\mathrm{IC}}_{2}$, slightly inferior results are obtained compared to ${\mathrm{IC}}_{3}$, but with a much higher ${\mathrm{FoM}}_{IC}$ value and better power consumption. On the other hand, comparing ${\mathrm{IC}}_{1}$ to ${\mathrm{IC}}_{2}$, the improvement in ${\mathrm{FoM}}_{IC}$ or power consumption is not as significant, meaning ${\mathrm{IC}}_{2}$ is not as efficient as ${\mathrm{IC}}_{1}$, yet the results are inferior to state-of-the-art K-band LNAs. In the case of ${\mathrm{IC}}_{3}$, the results can be further improved, but with a strong impact on the LNA’s power consumption with small gains in performance. Ultimately, the decision is to proceed with the layout design for ${\mathrm{IC}}_{2}$ = 1.76 μA, as it provides enough gain and NF to be in line with state-of-the-art solutions with the best trade-off between performance and power consumption, which will be explained in more detail in the following section.

(9) $${\mathrm{FoM}}_{IC}=\frac{2\cdot S_{21}\left[dB\right]}{NF\left[dB\right]\cdot P_{DC}\left[mW\right]}$$

After assembling the schematic, the DC operating point of the circuit is analyzed to verify the previously calculated values. There is an expected deviation in the DC parameters, since the FETs were simulated in a common-source configuration with a $V_{DS}$ of 0.9 V. However, in the cascode the effective $V_{DS}$ for each FET is reduced to ∼0.45 V, and due to the nature of short-channel devices, there is a drain current mismatch. Therefore, $V_{GS}$ must be adjusted to ensure the drain current targeted value. Now, the LNAs’ matching components can be calculated. To bring the $Z_{11}$ and $Z_{opt}$ to the center of the Smith chart, the values of $L_{source}$, $L_{gate}$, and $L_{drain}$ inductors (see Figure 2) are calculated. Additionally, a $C_{out}$ value of 50 fF has been established for all three cases to improve the $S_{22}$.

## 3. MOSFET Characterization and Simulation Results

To include the effect of metal interconnections on FET performance early in the design process, the layout of the cascode is developed first [40]. The new component consists of the raw PDK FET device, an RCC (resistance, capacitance, and coupled capacitance) parasitic extraction of the low-level, thin metal layers, and an EM characterization of the high-level, thick metal layers to account for parasitic inductance. As indicated in expression (10), the value of ${\mathrm{NF}}_{min}$ can be optimized [38]. Note that $R_{G}$ is the gate resistance, $R_{S}$ is the source resistance, *f* is the working frequency, and $f_{T}$ is the unity current gain frequency.

(10) $${\mathrm{NF}}_{min}=1+K\cdot \sqrt{\left(g_{m}\cdot \left(R_{G}+R_{S}\right)\right)}\cdot \frac{f}{f_{T}}$$

**Figure 7:**
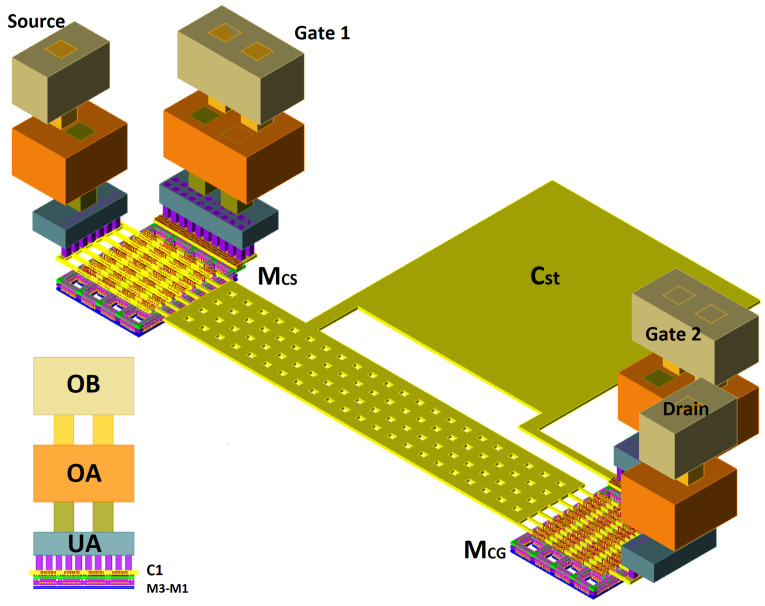
3D view of the two MOSFETs used to design the cascode LNA.

The circuit is implemented using the GlobalFoundries 45 nm RFSOI PDK. The technology has seven copper (Cu) layers (M1-M3, C1, UA, OA, OB) and one aluminum (Al) layer with a thickness of 4.125 μm. The RCC extraction is obtained using Calibre xRC extraction, and the EM characterization is obtained from EMX Planar 3D software. A staircase configuration is used to optimize the NF (${\mathrm{NF}}_{min}$) of this device by reducing parasitic capacitances and gate and source resistances. To facilitate DRC rules compliance and current flow, the FETs are divided into four instances. In addition, a $C_{GS}$ capacitor ($C_{st}$ in Figure 2) is added in metal layer C1 to improve device stability. The 3D view of the developed layout is presented in Figure 7.

**Figure 8:**
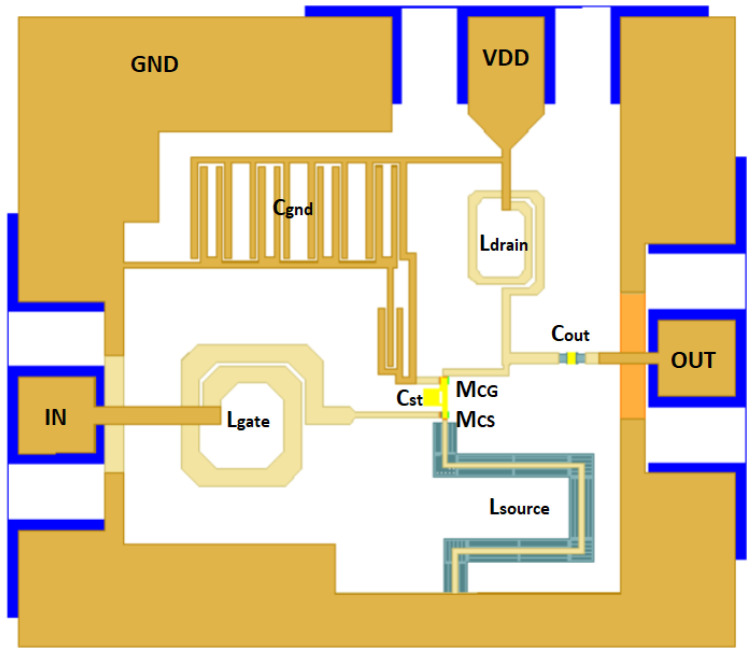
Simplified frontal view of the cascode LNA final layout.

Once the MOSFET characterization is availablem the schematic in Figure 2 is set up and the values of the inductors needed are adjusted to compensate for the deviations introduced by the new RCC + EM characterization of the cascode MOSFETs. The gate inductor is adjusted to 409 pH to achieve input impedance matching, $L_{drain}$ is reduced to 313 pH, $L_{source}$ is reduced to 130 pH and substituted by a transmission line, and $C_{out}$ is adjusted to 24 fF to improve $S_{22}$. In addition, for proper AC grounding, a number of 1 pF shunt capacitors are added at the gate of $M_{CG}$. The final cascode layout is shown in Figure 8.

**Figure 9:**
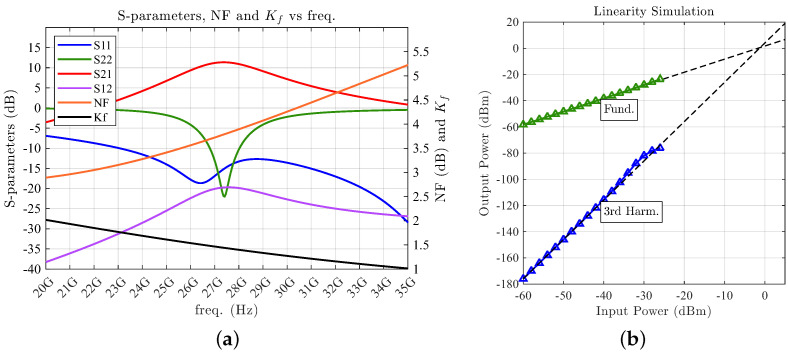
Simulation results of the proposed LNA with the EM characterization of each passive component (**a**) and two-tone linearity simulation for the obtainment of the IIP3 (**b**).

A post-layout extraction of the S-parameters after EM simulation with all the passive components in the final circuit is performed to verify the LNA. The simulation results are presented in Figure 9, showing a gain of 11.4 dB and a NF of 3.8 dB at a central frequency of 28 GHz. Regarding the input and output return losses, an |$S_{11}$| of 12.7 dB and an |$S_{22}$| better than 10 dB are obtained. The LNA draws a total of 1.98 mW from a 0.9 V DC supply and occupies a core area of 0.723 × 0.598 mm^2^. As shown in Figure 9b, a two-tone simulation with 100 MHz spacing is conducted to verify the LNA’s linearity, demonstrating a P-1 dB of 1.8 dBm and an IIP3 of −1 dBm.

Table 2 presents a comparison with some of the most relevant state-of-the-art solutions. In [41], the authors develop a mm-wave multi-band LNA in 45 nm CMOS SOI using a three-stage differential cascode with interstage transformer-based matching networks to save area. It achieves a notable gain of 19.5 dB with an NF of 4.7 dB, but at the cost of high power consumption (59 mW). A single-ended-input, differential-output tunable K/Ka-band LNA operating at 28 and 39 GHz is demonstrated in a 65 nm CMOS in [42], maintaining a very low NF of 2.8 dB with a gain of 17.2 dB, yet with a very high power consumption of 28.5 mW. In [43], a 22 nm CMOS fully depleted-SOI low-power LNA with a single-stage cascode configuration is presented. It achieves a remarkable power consumption of only 4.6 mW, a gain of 7 dB, and an NF of 5 dB. A dual-band LNA in 22 nm CMOS FDSOI for 5G wireless systems is demonstrated in [44]; the authors report a high gain (19.3 dB) and a minimum NF of 5.2 dB with a power consumption of 11.4 mW in 0.27 mm^2^. The LNA achieves simultaneous dual-band operation by employing a two-stage single-ended cascode topology with carefully optimized transmission lines and capacitor-based matching networks. In [29], a wideband (14 and 31 GHz) LNA in 54 nm CMOS SOI is demonstrated, providing a low NF of only 1.4 dB with 12.8 dB gain. However, this LNA draws as much as 15 mW and occupies an area of 0.3 mm^2^. In contrast, we propose a low-power LNA that achieves a gain of 11.4 dB with only 1.98 mW power consumption, while maintaining an NF within the average range of the other state-of-the-art designs. The developed work demonstrates the $g_{m}/I_{D}$ methodology can effectively be used to obtain an LNA with a gain above 10 dB with a low NF. We report a very low power consumption with remarkably high performance at Ka-band frequencies.

## 4. Conclusions

The design of a low-power Ka-band cascode LNA using the $g_{m}$/$I_{D}$ methodology is discussed in this work. The proposed circuit achieves a very low power consumption of only 1.98 mW. In addition, it is the first time the $g_{m}$/$I_{D}$ approach is applied at Ka-band frequencies. The proposed circuit is developed with the 45 nm SOI PDK components. The LNA has a central frequency of 28 GHz as the circuit operates in the n257 frequency band defined by 3GPP NR for European mm-wave communications. The design approach presented involves the $g_{m}$/$I_{D}$ methodology to exploit the advantages of sub-threshold operation in a high-frequency scenario, biasing devices in the moderate inversion region for a remarkable performance trade-off. A single MATLAB script is used to generate all the sweeps and the simulation netlists, to run the SPECTRE simulations, and to map the desired parameters in a multidimensional .MAT file containing all the LUTs needed to perform automated circuit design. We explore the procedure by providing a design example, from scratch to post-layout simulations, of a Ka-band cascode LNA obtaining a very-low-power, high-performance amplifier. The final circuit draws 1.98 mW from a DC supply of 0.9 V with a chip size of 0.43 mm^2^ excluding pads. After post-layout parasitic extraction and EM analyses, the circuit exhibits a gain of 11.4 dB, an NF of 3.8 dB, and an IRL better than 12 dB across the band of interest. Finally, a comparison is made with similar works available in the literature. The proposed circuit shows a very high performance, since the amplifier obtains a gain and NF in line with other works and a very low power consumption of only 1.98 mW. 

## Figures and Tables

**Table 1:** Values of the DC and AC parameters of the three selected ICs.

Parameter	${\mathrm{IC}}_{1}$ = 0.48	${\mathrm{IC}}_{2}$ = 1.58	${\mathrm{IC}}_{3}$ = 4.92
$V_{GS}$ (mV)	182	255	360
$V_{TH}$ (mV)	94	88	76
$I_{D}$ (mA)	0.67	2.2	6.8
$g_{m}$ (mS)	12	28.7	55.2
$g_{m}$/$I_{D}$ (S/A)	18	13	8
W/L	1.25 k	1.25 k	1.25 k
${\mathrm{NF}}_{min}$ (dB)	3.9	1.9	1.4
$G_{max}$ (dB)	8.6	14.2	17
$P_{DC}$ (mW)	0.6	1.98	6.2
$L_{source}$ (pH)	180	135	104
$L_{gate}$ (pH)	458	408	352
$L_{drain}$ (pH)	500	500	500
${\mathrm{FoM}}_{IC}$	5.7	4.2	2

**Table 2:** Overview of similar state-of-art LNAs with the proposed circuit.

Reference	This Work	[41]	[42]	[43]	[44]	[29]
Tech.	45 nm	45 nm	65 nm	22 nm	22 nm	45 nm
	SOI	SOI	CMOS	SOI	SOI	SOI
BW (GHz)	25.5–29.5	21–28	23.5–32.5	24–28	26.6–31.6	14–31
Centre Freq. (GHz)	27.5	24.5	27.5	26	29.1	22.5
Supply (V)	0.9	1	1	0.8	1.6	1.5
Max Gain (dB)	11.4	19.5	17.2	7	19.3	12.8
Threshold Gain (dB)	8.4	16.5	14.2	4	16.3	9.8
NF (dB)	3.5–3.8	4.7	2.8–3	5	5.2	1.4
IRL (dB)	12.7	–	25	6	10	10
ORL (dB)	10	–	–	–	10	10
$P_{DC}$ (mW)	1.98	59	28.5	4.6	11.4	15
Meas./Sim.	Sim	Meas.	Meas.	Meas.	Meas.	Meas.
${\mathrm{FoM}}_{IC}$	3.03	0.14	0.4	0.608	0.65	2.08
Core area (mm^2^)	0.43	0.42	0.157	0.1	0.27	0.3

## Data Availability

Data is contained within the article.

## Abbreviations

The following abbreviations are used in this manuscript:

$C_{gs}$	Gate-Source Capacitance
$C_{gd}$	Gate-Drain Capacitance
$C_{sb}$	Source-Drain Capacitance
$C_{db}$	Drain-Bulk Capacitance
CMOS	Complementary Metal Oxide Semiconductor
IC	Inversion coefficient
$I_{D}$	Drain current
$I_{GD}$	Gate-Drain Current
$I_{GS}$	Gate-Source Current
IRL	Input Return Loss
FB	Floating-Body
FoM	Figure of Merit
$g_{ds}$	Drain-Source Transconductance
$g_{m}$	Regular Transconductance
$g_{mb}$	Gate-Bulk Transconductance
LNA	Low-Noise Amplifier
MMIC	Monolithic Microwave Integrated Circuit
MW	Microwave
NF	Noise figure
ORL	Output Return Loss
PDK	Process Design Kit
RF	Radiofrequency
RFIC	RF Integrated Circuit
UWB	Ultrawide Band
VDS	Drain-Source Voltage
VGA	Variable Gain Amplifier
VGS	Gate-Source Voltage
VTH	Threshold Voltage
SATCOM	Satellite Communications
SOI	Silicon-on-insulator
STH	Thermal Noise coefficient
SFL	Flicker Noise Coefficient
$w_{f}$	Unit Finger Width
